# Severe IL-6–dominant immune-mediated inflammatory response complicated by probable arginine vasopressin deficiency following ivonescimab-based therapy in advanced EGFR-mutant lung squamous cell carcinoma: a case report

**DOI:** 10.3389/fonc.2026.1836652

**Published:** 2026-05-25

**Authors:** Fang Yang, Linli Xuan, Jun Li

**Affiliations:** 1 Department of Oncology, Jiahui International Hospital, Shanghai, China; 2 Department of Intensive Care Unit (ICU), Jiahui International Hospital, Shanghai, China

**Keywords:** arginine vasopressin deficiency, EGFR mutation, hypophysitis, IL-6–dominant immune-mediated inflammatory response, Ivonescimab, non-small cell lung cancer, PD-1/VEGF bispecific antibody, PD-L1

## Abstract

**Background:**

Ivonescimab, a novel PD-1/VEGF bispecific antibody, has shown promising efficacy in advanced lung cancer. Yet severe immune-related adverse events are not well studied outside clinical trials.

**Case presentation:**

We report a 72-year-old man with stage IV squamous NSCLC harboring an EGFR mutation and PD-L1 tumor proportion score of 90% who developed persistent fever shortly after first exposure to ivonescimab combined with nab-paclitaxel and carboplatin. His prior treatments included concurrent chemoradiotherapy, osimertinib, the investigational EGFR inhibitor BH-30643, afatinib, and recent gamma knife for cerebellar metastases plus palliative radiotherapy for bone metastases. After cycle 1, he developed persistent high fever with markedly elevated IL-6 and no response to antimicrobials. Corticosteroids brought rapid relief, pointing to an IL-6–mediated process. Re-exposure to ivonescimab triggered a more severe reaction. He then developed sudden polyuria with low urine osmolality and elevated serum osmolality. Brain MRI showed no typical hypophysitis features. Symptoms improved with corticosteroids, tocilizumab, and desmopressin, consistent with probable arginine vasopressin deficiency (AVP-D), possibly secondary to hypophysitis—though pituitary metastasis cannot be ruled out without biopsy.

**Conclusions:**

Ivonescimab can trigger severe IL-6–dominant inflammatory reactions and endocrine toxicity presenting as AVP-D in susceptible patients. Advanced age, EGFR-mutant/PD-L1-high tumor biology, recent radiotherapy, and Parkinsonism may have contributed to the inflammatory environment, though none can be proven causal in a single case and each deserves prospective study. Early recognition of persistent steroid-responsive fever, serial IL-6 monitoring, thorough endocrine workup, and timely IL-6 blockade are critical.

## Introduction

Immune checkpoint blockade has transformed NSCLC treatment, but irAEs remain a significant clinical concern and can be life-threatening (1). Endocrine irAEs typically affect the thyroid and pituitary; AVP-D is rare (2).

Ivonescimab is a PD-1/VEGF bispecific antibody that outperformed pembrolizumab in PD-L1 positive NSCLC (HARMONi-2) and tislelizumab plus chemotherapy in advanced squamous NSCLC (HARMONi-6), with a generally manageable toxicity profile (3, 4). However, severe inflammatory syndromes may go unreported outside trial summaries (5).

Here we describe an elderly patient with EGFR-mutant, PD-L1 high squamous NSCLC who developed a severe steroid-responsive IL-6–dominant inflammatory response followed by probable AVP-D after ivonescimab-based chemotherapy.

## Case presentation

A 72-year-old man was diagnosed in July 2023 with left lower lobe squamous cell carcinoma, clinical stage IIIA (cT3N1aM0), with an EGFR mutation and 90% PD-L1 expression. He received weekly carboplatin/paclitaxel concurrent chemoradiotherapy from August 7 to September 25, 2023, then osimertinib 80 mg daily from December 2023. After progression, he enrolled in a trial of the next-generation EGFR inhibitor BH-30643 on June 5, 2025, discontinuing on July 11, 2025, due to toxicity. Afatinib 20 mg daily was started September 6, 2025. PET-CT on November 6, 2025, showed multiple bone and lymph node metastases; disease was progressive. On November 11, 2025, he received gamma knife for cerebellar metastases and palliative radiotherapy for bone metastases.

On November 27, 2025, he began ivonescimab plus chemotherapy: ivonescimab 1280 mg (20 mg/kg) day 1, nab-paclitaxel 200 mg day 1 and 100 mg day 8, carboplatin 247 mg (AUC 2.5) days 1 and 8, every 21 days. On November 30, 2025, he developed chills and fever to 39 °C. Influenza A and B IgM were positive, but whether this reflected active infection or prior exposure was unclear. Antivirals were given without effect. Blood and sputum cultures were negative, urinalysis normal, chest CT showed no pneumonia. Antibiotics failed, and temperature held around 38 °C. Day-8 chemotherapy was held.

On December 18, 2025, IL-6 was 59.85 pg/mL (reference 0–7 pg/mL) (5). MetaCAP blood sequencing detected CMV DNA. Ganciclovir 300 mg IV q12h and immunoglobulin 10 g daily were started. After 3 days, fever persisted; quantitative CMV-DNA was <4.00E+02 copies/mL, making active CMV disease unlikely (1). The clinical presentation-persistent fever despite negative cultures, no radiographic pneumonia, unresponsive to antimicrobials, and rapid resolution with steroids, strongly suggested an immune-mediated etiology. Methylprednisolone 40 mg IV bid was started December 22, 2025; temperatures gradually fell. By December 29, IL-6 had dropped to 3.98 pg/mL, and steroids were reduced to 40 mg daily.

**Figure 1:**
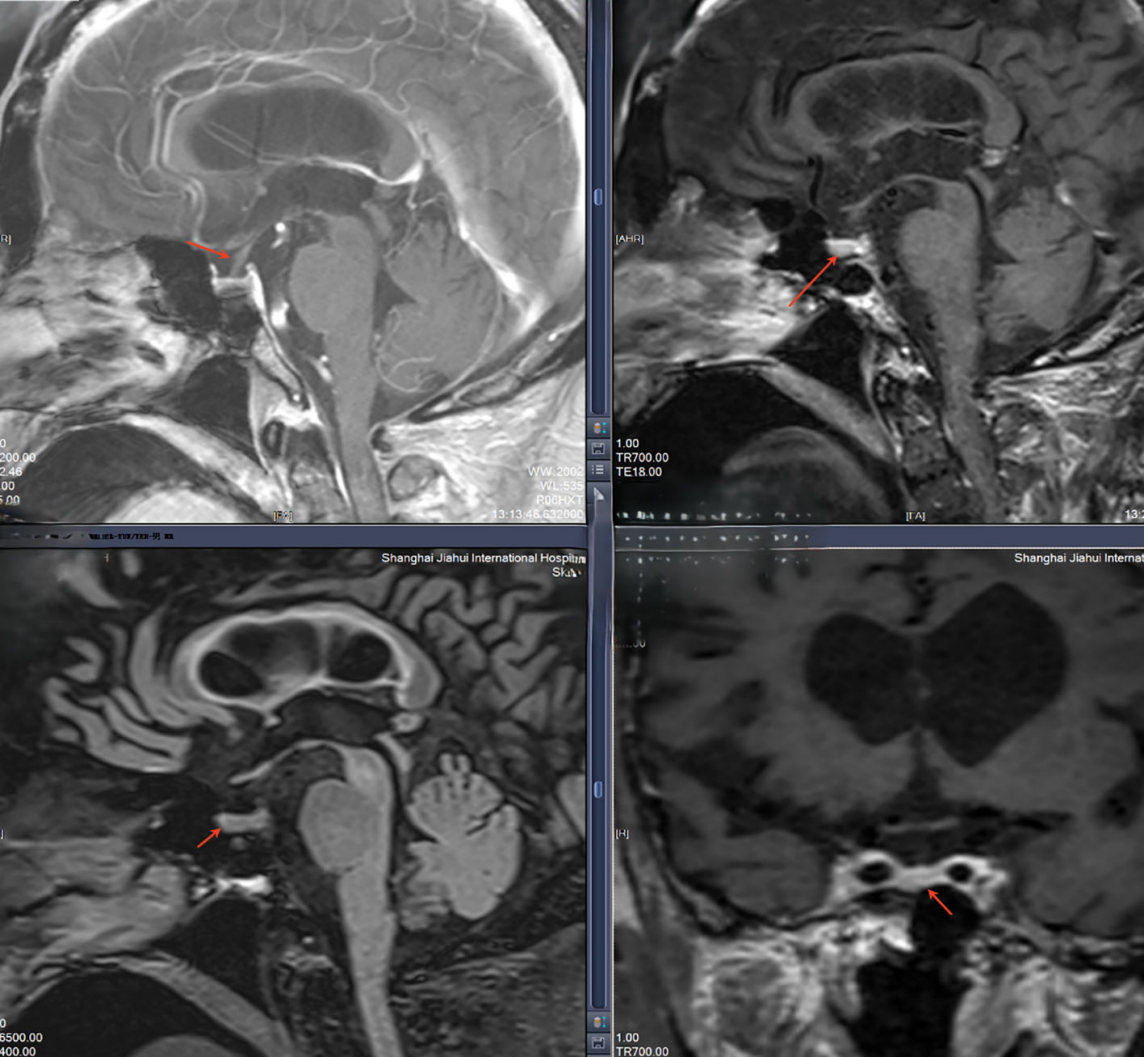
January 12, 2026 Brain MRI showed slight pituitary stalk thickening.

Cycle 2 was given December 30, 2025: ivonescimab 1280 mg, nab-paclitaxel 460 mg, carboplatin 522 mg on day 1. On January 6, 2026, grade 4 myelosuppression developed (neutrophil count 0.05 × 10^9^/L) with fever to 38.9 °C. Chest X-ray remained clear. IL-6 increased to 622.00 pg/mL. Due to previous steroid-responsive episode and the absence of an infectious source, severe immune-mediated toxicity was presumed (5). The patient received G-CSF, meropenem, and methylprednisolone 60 mg IV. Tocilizumab 240 mg (4 mg/kg) was given January 8 and 9, 2026; IL-6 fell to 193.20 pg/mL and temperature stabilized at 37.2–37.5 °C (6) (**Figure 1**).

On January 9, 2026, abdominal distension prompted bedside ultrasound showing 1300 mL bladder residual without ascites. After catheterization, 5600 mL drained in 4 hours. Urine osmolality was 241 mOsm/kg (reference 600–1000), serum osmolality 313 mOsm/kg (reference 275–305), serum sodium 145 mmol/L (reference 135–147). AVP-D was suspected (2). He immediately became hypotensive with atrial fibrillation, requiring ICU transfer. Vasopressin, fluids, and amiodarone stabilized him.

**Figure 2:**
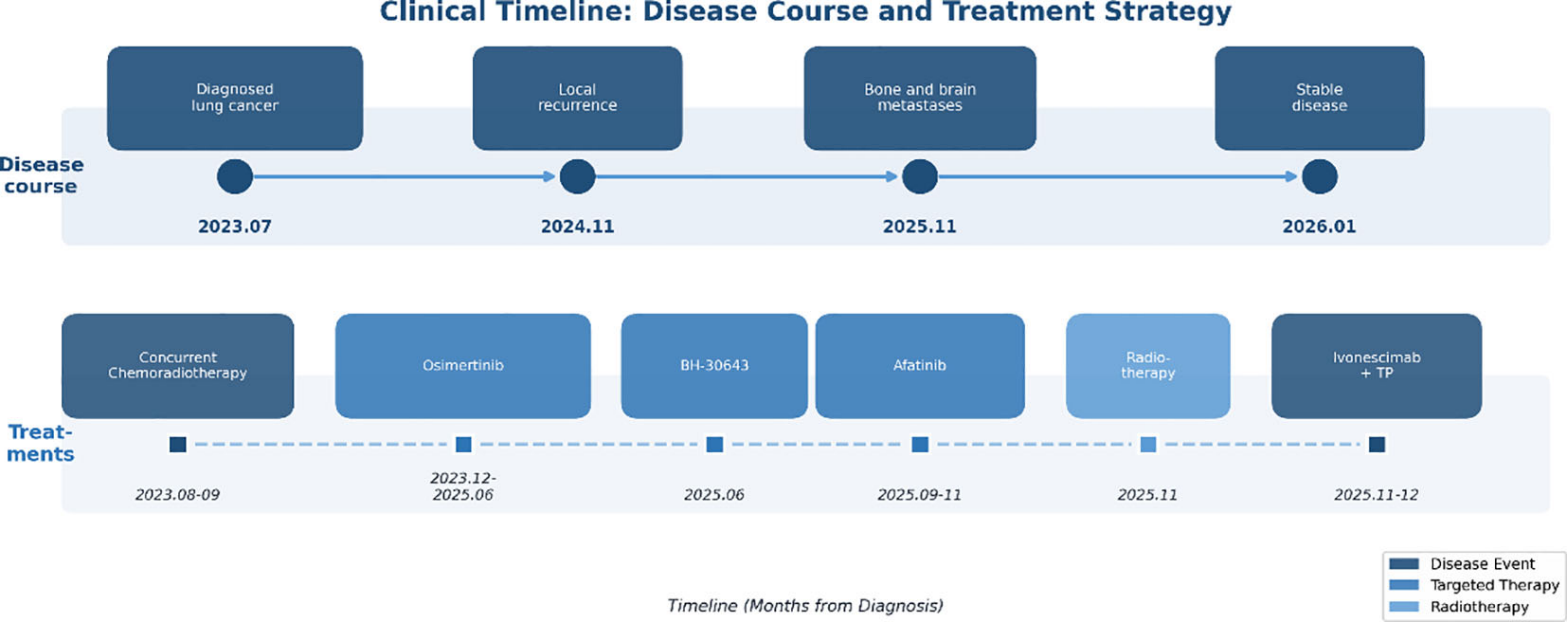
Timeline of lung cancer course and treatments.

**Figure 3:**
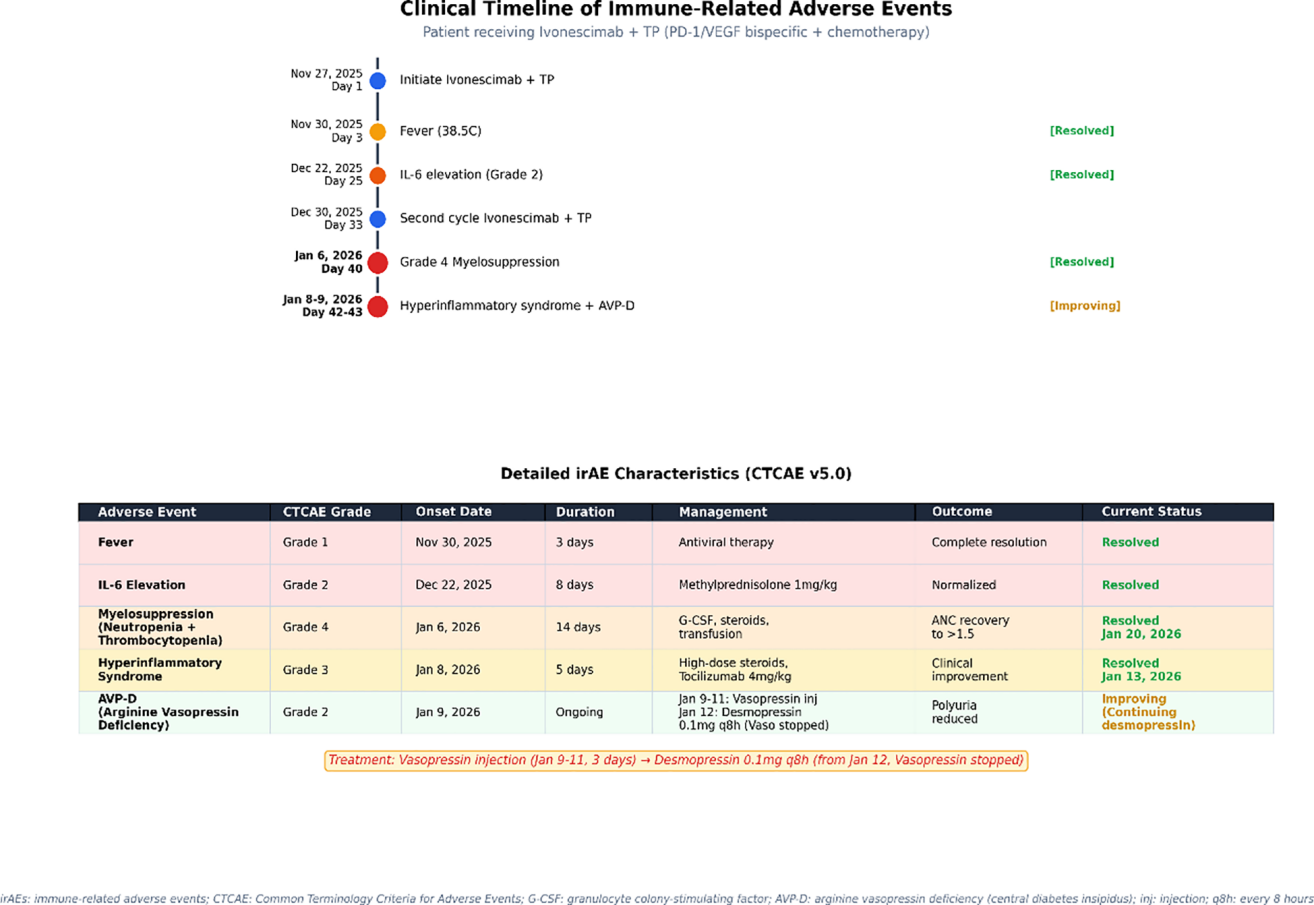
Clinical timeline of immune-related adverse events.

**Figure 4:**
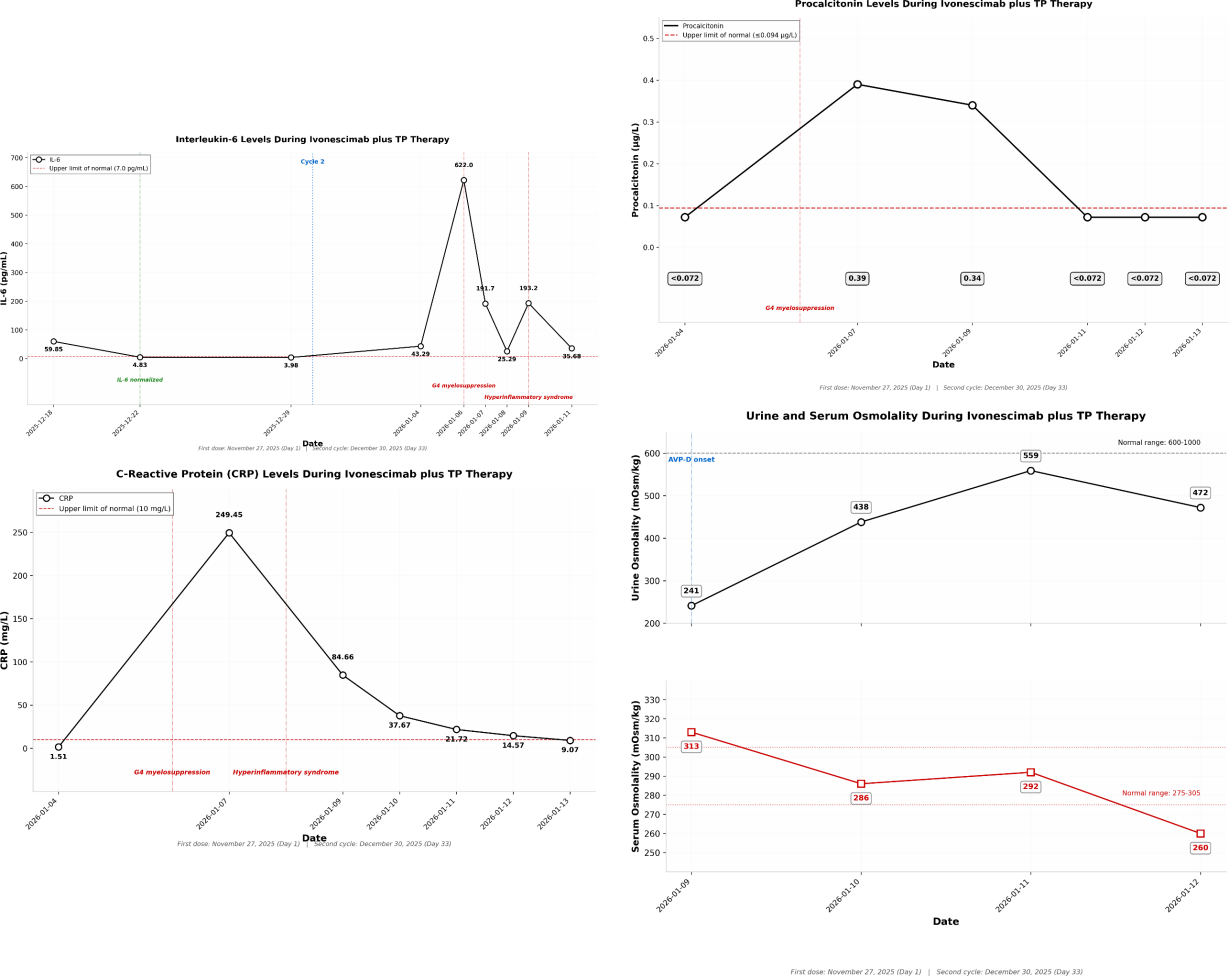
Dynamic changes in interleukin-6 (IL-6), C-reactive protein, procalcitonin, urine osmolality and serum osmolality levels in relation to immunotherapy and interventions.

By January 11, 2026, IL-6 was 35.68 pg/mL (**Figure 2**). On January 12, urine specific gravity improved; he was switched to oral desmopressin 0.1 mg q8h (**Figure 3**). Brain MRI that day showed stable or smaller intracranial metastases and no characteristic hypophysitis features (7) (**Figure 4**).

### Differential diagnosis of AVP-D etiology

The onset of AVP-D was 6 weeks after the first ivonescimab dose. ICI-hypophysitis typically presents at 6–12 weeks with isolated ACTH deficiency (2, 7), rather than isolated AVP-D. This relatively early onset, in a patient with stage IV lung cancer and known brain metastases, raises pituitary metastasis as an important alternative (7). Pituitary metastases from lung cancer may present with AVP-D and are radiologically indistinguishable from hypophysitis without biopsy (7).

Our endocrine workup was incomplete. Notably, ACTH was 28.4 ng/L (reference 7.2–63.3), but serum cortisol was markedly elevated at 819 nmol/L (reference 68.2–327.0) on January 7, likely reflecting acute stress response. Without a formal cosyntropin stimulation test or baseline morning cortisol prior to steroid administration, we cannot exclude secondary adrenal insufficiency. The normal ACTH level in this setting does not argue against hypophysitis; rather, the pituitary-adrenal axis was not fully characterized. We therefore classify this as “probable AVP-D, etiology uncertain (hypophysitis versus pituitary metastasis)” (2).

Picture 1. January 12, 2026 brain MRI showed slight pituitary stalk thickening.

## Discussion

This patient experienced two distinct but likely related toxicities from ivonescimab: a severe recurrent IL-6–dominant systemic inflammatory response, and probable AVP-D with partial anterior pituitary dysfunction (5).

IL-6–dominant immune-mediated inflammatory response. The inflammatory was characterized by persistent high fever, a significant increase in IL-6 levels (peak 622 pg/mL), and rapid clinical deterioration on rechallenge. The fever was unresponsive to antivirals or antibiotics but promptly resolved with corticosteroids and tocilizumab, providing strong evidence of an immune-mediated mechanism (5). The second episode proved worse than the first, with grade 4 myelosuppression and hemodynamic instability requiring ICU admission, consistent with immune-mediated toxicity (1). Tocilizumab produced rapid clinical improvement, highlighting IL-6 as the central player (6).

We avoid labeling this “cytokine release syndrome (CRS).” The ASTCT 2019 grading criteria were developed for hematologic malignancies and T-cell–engaging therapies and do not applicable to solid tumor bispecifics like ivonescimab, which lacks a validated CRS framework. The patient met criteria for severe systemic inflammation—fever >38 °C, marked IL-6 rise, hemodynamic instability, grade 4 myelosuppression—but hypoxia was absent (8).

Infection versus immune-mediated toxicity. Influenza A/B IgM positivity and MetaCAP-detected CMV DNA initially raised infection concerns (1). Yet the overall clinical presentation suggested immune-mediated toxicity: fever within 72 hours of ivonescimab; negative cultures and imaging; antimicrobial failure; rapid response to steroids; and more severe recurrence on rechallenge (5). MetaCAP detects viral nucleic acids but cannot separate active replication from latent shedding; quantitative CMV-DNA was low (<4.00E+02 copies/mL) (1). Without organ-specific disease or rising viremia, CMV as the primary driver was improbable. The influenza serology likely represented prior exposure rather than active infection, as viral PCR was unavailable. In ICI-treated patients, such findings should be interpreted with caution and not allowed to distract from the characteristic pattern of immune-mediated toxicity (1).

AVP-D: hypophysitis or pituitary metastasis? The diagnosis stays at “probable.” Current endocrine irAE guidelines require histopathological confirmation or a classic clinical-radiological picture (2). What we have instead is atypical: relatively early onset (6 weeks), AVP-D as the presenting feature rather than isolated ACTH deficiency, and MRI without characteristic hypophysitis features (7). Moreover, the endocrine evaluation was incomplete, with some measurements complicated by acute stress and corticosteroid administration. Distinguishing between pituitary metastasis from lung cancer and hypophysitis is crucial, with biopsy being the definitive diagnostic method (2). As noted above, the lack of a complete baseline cortisol assessment limits interpretation of the normal ACTH value. The typical presentation of ICI-induced hypophysitis begins with isolated ACTH deficiency; our inability to fully assess the pituitary-adrenal axis means this characteristic feature could not be confirmed or excluded. We therefore classified this as “probable AVP-D, etiology uncertain (hypophysitis versus pituitary metastasis).”

What may have contributed. Advanced age (72) probably reduced physiological reserve, explaining the hemodynamic instability and ICU course, but age alone is an inconsistent predictor of irAE risk (9). EGFR-mutant NSCLC carries an immunologically “cold” microenvironment and responds poorly to PD-1/PD-L1 monotherapy (10); however, EGFR signaling upregulates PD-L1 independently of immune infiltration (11), and prior EGFR-TKI exposure may have altered tumor-immune interactions. Whether EGFR mutation predisposes to severe irAEs with bispecific antibodies needs prospective study. High PD-L1 (90%) in EGFR-mutant tumors may be driven by oncogenic signaling rather than immune engagement (11); its role in amplifying inflammation here remains speculative. Parkinson’s disease carries elevated IL-6 and altered T-cell function (12), which might lower the threshold for neuroendocrine immune toxicity, though direct evidence is lacking. Parkinsonism also complicated the clinical presentation, potentially obscuring early signs of hypophysitis or AVP-D (12). Recent gamma knife and palliative radiotherapy, completed just 16 days before ivonescimab, may have facilitated antigen release and disrupted the blood-brain barrier (13), potentially contributing to both systemic and central nervous system inflammation—though this remains conjecture.

Limitations. Several limitations of this case report should be acknowledged. First, the endocrine evaluation was incomplete: LH, FSH, IGF-1, and formal dynamic testing were not performed; cortisol was measured during acute stress (819 nmol/L on January 7) without cosyntropin stimulation assessment; and thyroid function testing was complicated by levothyroxine replacement. These gaps preclude definitive characterization of the pituitary-adrenal axis and limit our ability to distinguish hypophysitis from metastasis. Second, pituitary biopsy was not obtained, which is the only definitive method to differentiate immune-related hypophysitis from pituitary metastasis. Third, CMV IgM/IgG were not tested, and influenza PCR was unavailable, limiting our ability to definitively exclude viral infection. Fourth, this is a single case, and no causal inferences can be drawn regarding the contributions of age, EGFR mutation, PD-L1 expression, Parkinsonism, or radiotherapy to the observed toxicities.

Practical implications. Persistent fever after novel immunotherapy combinations should not be automatically attributed to infection when cultures are negative and imaging unrevealing (1). Serial IL-6 tracking can identify cytokine-driven processes and guide IL-6–directed therapy (5). Rechallenge after a severe steroid-responsive inflammatory event should be avoided or undertaken with extreme caution (1). Abrupt polyuria in an ICI-treated patient should trigger immediate AVP-D workup, including serum sodium, osmolality, and anterior pituitary hormones (2). Without histopathology, immune-related hypophysitis should remain a probable diagnosis, with pituitary metastasis actively considered in advanced lung cancer (7).

## Conclusion

This case illustrates that ivonescimab can trigger severe IL-6–dominant inflammatory reactions requiring tocilizumab alongside corticosteroids, and that AVP-D in this setting presents a diagnostic challenge between hypophysitis and pituitary metastasis that cannot be resolved without biopsy. The key learning is to maintain diagnostic caution when endocrine evaluation is incomplete and to prioritize IL-6 blockade for severe cytokine-driven toxicity.

## Data Availability

The original contributions presented in the study are included in the article/supplementary material. Further inquiries can be directed to the corresponding author.
